# From Normal Skin to Squamous Cell Carcinoma: A Quest for Novel Biomarkers

**DOI:** 10.1155/2016/4517492

**Published:** 2016-08-23

**Authors:** Vlad Voiculescu, Bogdan Calenic, Mihaela Ghita, Mihai Lupu, Ana Caruntu, Liliana Moraru, Suzana Voiculescu, Alexandra Ion, Maria Greabu, Nikolay Ishkitiev, Constantin Caruntu

**Affiliations:** ^1^Department of Dermatology and Allergology, Elias Emergency University Hospital, Bucharest, Romania; ^2^Department of Biochemistry, Faculty of Dental Medicine, “Carol Davila” University of Medicine and Pharmacy, Bucharest, Romania; ^3^Dermatology Research Laboratory, “Carol Davila” University of Medicine and Pharmacy, Bucharest, Romania; ^4^Department of Oral and Maxillofacial Surgery, “Carol Davila” Central Military Emergency Hospital, Bucharest, Romania; ^5^Department of Physiology, “Carol Davila” University of Medicine and Pharmacy, Bucharest, Romania; ^6^Department of Medical Chemistry and Biochemistry, Faculty of Medicine, Medical University, Sofia, Bulgaria

## Abstract

Squamous cells carcinoma (SCC) is the second most frequent of the keratinocyte-derived malignancies after basal cell carcinoma and is associated with a significant psychosocial and economic burden for both the patient himself and society. Reported risk factors for the malignant transformation of keratinocytes and development of SCC include ultraviolet light exposure, followed by chronic scarring and inflammation, exposure to chemical compounds (arsenic, insecticides, and pesticides), and immune-suppression. Despite various available treatment methods and recent advances in noninvasive or minimal invasive diagnostic techniques, the risk recurrence and metastasis are far from being negligible, even in patients with negative histological margins and lymph nodes. Analyzing normal, dysplastic, and malignant keratinocyte proteome holds special promise for novel biomarker discovery in SCC that could be used in the future for early detection, risk assessment, tumor monitoring, and development of targeted therapeutic strategies.

## 1. Introduction

Squamous cell carcinoma (SCC) is responsible for 20% of skin malignancies [1, 2]. Although most SCCs are curable, it was shown that 14% of them metastasize and of these unfortunate patients, 40% will eventually die; therefore they are responsible for the majority of deaths caused by nonmelanoma skin cancer [3–5]. Annually there are 400.000–600.000 new cases of cutaneous SCC (cSCC) diagnosed all over the world, more frequently among fair-skinned people. In USA and China studies show that 700.000 new cases of cSCC are diagnosed every year [6]. The incidence of skin cancer is growing with 5% every year in Central Europe and with 4% in regions with low sunlight exposure, such as Finland [7]. The major factor which influences the occurrence of skin abnormal cells which further evolve into cSCC is UV radiation, especially recreational sun exposure, which is in particular responsible for the increasing incidence of skin cancer in young people [8]. This is the reason why cSCC usually appears on the face and neck, sites that are frequently exposed to sunlight [9]. Other risk factors are exposure to carcinogenic chemicals, chronic skin ulceration, and immunosuppressive medication [1, 10, 11]. Actinic keratosis (AK) is a lesion that precedes cSCC, although not all AK progress into cSCC, which is represented by abnormal intraepidermal keratinocytes [1]. When these abnormal cells pass beyond the basement membrane we face an invasive cSCC, which has a greater risk of metastasis [12, 13]. Histopathological examination is considered the gold standard of diagnosis for SCC and other skin tumors, but noninvasive and minimal invasive diagnostic techniques have gained increased attention in the past years, as they do not imply performing a skin biopsy [14, 15]. Although there are efficient methods of treatment available for cSCC, none of them can assure a complete healing, thereby 8% of cSCCs recur and 5% metastasize within 5 years [1]. This is the reason why there is a high necessity of identifying molecules that can help evaluate the risk of recurrence and metastasis from early stage [16]. It was noted that the risk of metastasis and recurrence varies depending on localization, so cSCC localized on the lips or ears is correlated with a higher risk of invasion (10–25%); initial tumor size, >2 cm, has 15% chances of recurrence and 30% chances of metastasis; histological features are, for example, the speed of tumor growth, tumor depth > 4 cm, poor differentiation, and perineural invasion [1, 3, 17].

Considering the increasing incidence in cSCC and the risk of metastasis and recurrence, even in patients with negative histological margins and lymph nodes, it is necessary to identify circulating molecules that can help predict the prognosis/evolution of this pathology [18–24]. Proteomics represents a field of molecular biology which studies the protein expression of an organism/cell. It is very well documented that DNA has the necessary information to synthesize the whole set of proteins that a cell needs to survive. Modifications in signalling pathways induce changes in gene expression and the result is the alteration in protein levels, which can be objectified through proteomics [25, 26]. In order to understand this mechanism and the way it correlates with different pathologies, clinical proteomics studies the characteristics of a specific protein (quantity, variation in time, and interactions) obtained from various biological fluid or tissue specimens. This tool may be useful in the diagnosis, prognosis, and therapy monitoring in various malignancies [27–33].

## 2. Proteome of Normal and Inflammatory Keratinocytes

Generally all epithelia play a paramount protective role for their underlying tissue, being the first line of defense against many harmful exogenous agents and at the same time acting as a permeable barrier that prevents loss of body fluids. Epithelia are usually composed of several layers of cells, each with their own specific phenotypes, being characterized by different degrees of differentiation according to their placement within the thickness of the tissue [34]. Keratinocytes constitute the most representative population of the epidermis and the proliferating cell in nonmelanoma skin cancers.

Studying the proteins present in normal keratinocytes and the changes of their pattern that occur in inflammation or carcinogenesis may lead to identification of new therapeutic targets or new biomarkers valuable in early diagnosis and prognosis of skin cancer [35].

A recent* in vitro* study [36] has identified 50 proteins considered specific to keratinocytes, most of them directly related to keratinocyte physiology. Some of these proteins, such as alpha-2 macroglobulin-like protein-1, alpha-2 macroglobulin-like protein 2, and interferon regulatory factor 6 (IRF-6) are involved in keratinocyte proliferation and differentiation. Another category of keratinocyte proteins that have attracted attention are dermokine and calmodulin-like protein 5, which are keratinocyte differentiation markers, and integrin beta 4, which plays a role in keratinocyte motility.

The same study revealed a change in keratinocyte pattern of proteins in inflammatory conditions induced by stimulation with IL-1 beta. Thus, the level of proteins with roles in keratinocyte differentiation, such as alpha-2 macroglobulin-like protein-1, and the level of proteins involved in motility of keratinocytes such as integrin beta 4 were reduced. On the other hand, the presence of proinflammatory cytokines, such as IL-1F9 and IL-18, was observed. Moreover, stimulation with IL-1 beta increased the level of proteins involved in nuclear factor kappa B (NF-*κ*B) communication pathway, in angiogenesis, and of those with antiapoptotic effect. Similar changes were found in epidermoid carcinoma cells suggesting an important role of inflammation in skin carcinogenesis [36].

## 3. Effects of Carcinogens on Keratinocyte Proteome

### 3.1. Ultraviolet Exposure Effects on Keratinocyte Proteome

**Figure 1:**
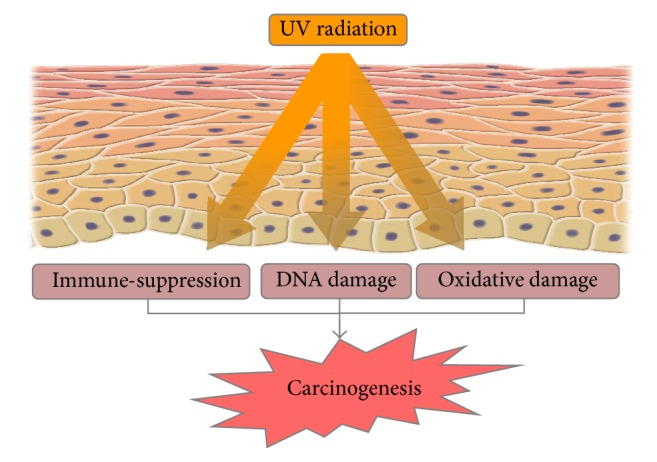
UV-induced skin carcinogenesis. UV radiation alters the normal immune responses, induces DNA damage and oxidative stress, and may lead to development of skin cancer.

UV exposure is one of the most important risk factors of skin cancer. Several studies using proteomic approaches have highlighted the alterations of protein expression induced by UV radiation on skin cells. UV exposure of skin induces suppression of cell-mediated immune responses, DNA damage, and formation of reactive oxygen species which can lead to oxidative stress and cellular damage (see Figure 1) [37, 38]. The first event after exposure to high doses of UV radiation is induction of keratinocyte apoptosis mediated by the p53/p21/bax/bcl-2 pathway and impairment of protein production, followed by hyperproliferation which may lead to subsequent epidermal hyperplasia [39, 40].

Chronic exposure to low doses of UV radiation also impacts the skin pattern of proteins by activation of different cellular signalling pathways, such as the mitogen-activated protein kinases (MAPK) pathway, the phosphoinositide 3-kinase (PI-3K) pathway, and the nuclear factor NF-*κ*B pathway, involved in modulation of cell growth, differentiation, proliferation, and motility. UV exposure increases expression of several matrix metalloproteinases (MMPs), such as MMP-1, MMP-3, and MMP-9, and the keratinocyte content of keratins 6, 16, and 17. On the other hand, it reduces type I collagen synthesis and impairs the transforming growth factor (TGF) beta communication pathway [38, 41–43]. Some of these changes may be associated not only with abnormal skin conditions, skin inflammation, but also with photoaging and skin carcinogenesis and the main challenge of future proteomic studies will be to identify a panel of biomarkers which allows differentiation between these various skin conditions.

### 3.2. Keratinocyte Proteomics in Chemically Induced Carcinogenesis

Exposure to carcinogenic chemicals is another factor that increases the risk of developing SCC. One of the main environmental factors with a strong link to skin carcinogenesis is arsenic [44]. Proteomic analysis of* in vitro* arsenic exposure of human keratinocytes showed a modified pattern of proteins with increased expression of several proteins such as heterogeneous nuclear ribonucleoprotein L isoform A, keratin 7, and keratin 9 [45] that can be associated with the development of premalignant lesions, or even SCC [46, 47]. On the other hand, in keratinocytes exposed to arsenic expression of involucrin was decreased, a similar pattern being previously highlighted in human cSCC [48].

Mouse models are the most commonly used animal models for the study of skin cancer, because in many aspects they mirror the mechanisms of human carcinogenesis [35].

However, there are numerous differences between distinct strains and different experimental models and there is hope that proteomic techniques will allow highlighting of the intimate mechanisms underlying these differences. Proteomic analysis in animals C57BL/6-resistant and DBA/2 sensitive, following 12-O-tetradecanoylphorbol-13-acetate (TPA) administration, demonstrated 19 different expressed proteins, such as S100 calcium binding proteins A8, A9, and A11 as well as parvalbumin *α* and annexin A1 [49]. After topical application of carcinogenic promoters chrysarobin and okadaic acid S100 proteins A8 and A9 were also elevated. Further research identified the majority of these proteins to be related to inflammation and more specifically to inflammatory networks that regulate and promote tumoral growth in skin such as TNF*α* and nuclear factor (NF)*κ*B. Moreover, after TPA exposure DBA/2 mice but not C57BL/6 mice express mRNAs for a wide array of inflammatory proteins, such as TNF, Nf*κ*b1, IL-22, and IL-1b, and chemokines such as Cxcl1, Cxcl2, and Cxcl5. Taken together, these results suggest that chemically induced carcinogenesis in murine models may be sustained by inflammatory genes activity [49]. Other proteins involved in skin carcinogenesis are cell surface markers such as tetraspanins, found on virtually all cell types [50]. CD markers are known to be expressed in several types of cancer; of these, CD151 has been shown to induce skin chemical carcinogenesis and to promote a fast development of SCC in mouse models. These results also match the results found in human SCC. CD151 is most often associated with activation of the transcription factor, signal transducer, and activator of transcription 3 (STAT3). The data suggests that CD151 may be used as a future antitumoral therapeutic target [51]. Other studies demonstrate that, in a murine carcinogenic model, DMBA-induced carcinogenesis in PKC*α* knockout mice tumor formation is suppressed but not tumor growth and progression [52]. Proteomics is a rapidly developing field that brings vital inputs in identifying and quantifying the proteome responsible for initiation and development of the carcinogenic process in skin [53].

## 4. Proteic and Other Potential Biomarkers of Squamous Cell Carcinoma

### 4.1. Cutaneous SCC

#### 4.1.1. Inflammatory Markers

Inflammation is involved in many types of pathologies, from AK and Bowen's disease (BD) to cutaneous SCC (cSCC) and other kind of cancers; thus the involvement of inflammatory markers, such as the complement factor H (CFH) and FHL-1 (factor H-like protein-1) in the development of cSCC has attracted an increasing interest [60]. CFH is a soluble molecule that has a role in inhibiting one of the three pathways which activates the complement C3, the alternate pathway (which is continuously activated* in vivo*), and it also represents a cofactor for complement factor 1 in the inactivation process of C3b to iC3b [61, 62, 97]. CFH exists as two molecules with largely the same functions: CFH (150 kDa) and factor H-like protein-1 (45 kDa) [98, 99]. Studies show that as the cell progresses from AK to cSCC it has a higher rate of expression of CFH, FHL-1, and complement factor 1 in cSCC cells [60]. Also CFH facilitates proliferation and migration of cSCC cells; thus it is associated with negative prognosis in patients with CFH overexpression. In the study conducted by Riihilä et al. [60], CFH and FHL-1 expressions were analyzed* in vivo* through qPCR of RNA, 6 samples from cSCC lesions, and 11 samples from normal skin, concluding that in cSCC lesions the expression of these molecules was significantly higher than in normal skin [60]. Tissue samples from AK, BD, and cSCC were analyzed through immunohistochemistry showing that the expression of these inhibitors increases with the progression of the lesion but it is present even in early stages, which makes detection of CFH and FHL-1 very useful [18]. The study of Riihilä et al. showed that cSCC cells express C3 more than normal keratinocytes, which may be the reason why the inflammatory reaction is important in cSCC, C3 being the main component which activates all three pathways of complement cascade. It was also noted that inflammatory cytokines like IFN-*γ*, IL-1*β*, and TNF-*α* increased the expression of CFH by cSCC cells. In cultures a significant quantity of iC3 was present, reflecting that cSCC cells produce active CFH that helps this cell population escape the complement mediated cell destruction, having a very important role in cSCC progression. It was also demonstrated that complement factor I degrades C3b into smaller molecules which facilitates CFH and FHL-1 activity [60, 100].

Serpin A1 or 1-antitrypsin is included in the serine peptidase inhibitors (Serpins) family which has a very large distribution in the human body and has various functions (coagulation, inflammation, and turnover of extracellular matrix). Serpins are divided into two groups: A which includes extracellular molecules and B formed by intracellular molecules [63, 64]. The value of Serpin A1 from cSCC samples was compared with the value from normal keratinocytes and it was noted that cSCC cells had a greater concentration and this result was correlated with the invasiveness and could be used for prognosis prediction. In addition, samples from AK were examined and it was observed that Serpin A1 was not as well expressed as in cSCC cells; this result pleads for the importance of Serpin A1 in detecting cSCC progression. It is well known that cSCC is accompanied by inflammation and studies show that inflammatory cytokines have tumor protective functions, theory supported by the fact that it was noted that the value of Serpin A1 is increased by TNF-*α*, IFN-*γ*, and IL-1*β* [113]. It has been demonstrated that Serpin A1 inhibits natural killer cell activity, stimulates malignant cell proliferation but not normal skin cell proliferation, and has an antiapoptotic effect (lung endothelial cells); therefore Serpin A1 has tumorigenic activity [101–103].

#### 4.1.2. Early Markers of Skin Carcinogenesis

**Figure 2:**
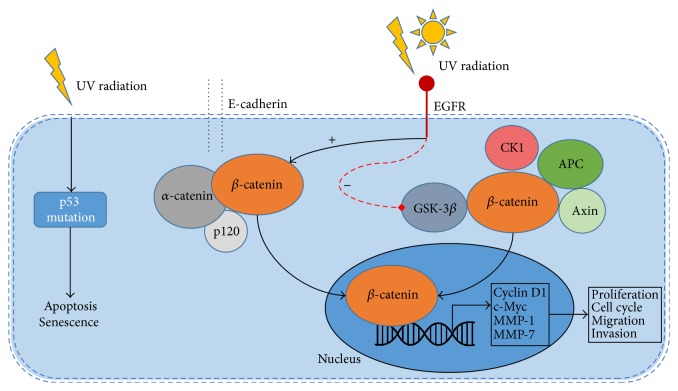
Dysregulation of cellular signalling in SCC. Aberrant activation of EGFR induces phosphorylation of *β*-catenin and GSK-3*β*, leading to uncoupling of *β*-catenin from both destruction complex (*β*-catenin/GSK-3*β*/APC/CK1/Axin) and E-cadherin/p120/*α*-catenin complex and translocation to the nucleus. Once translocated to the nucleus it influences gene transcription, including Cyclin D1, c-Myc, MMP-1, and MMP-7 (viable biomarkers for SCC) which have important roles in proliferation, cell cycle, migration, and invasion. The figure also shows one of the first events in SCC carcinogenesis, namely, the induction of tumor suppressor p53 mutations. ^*∗*^EGFR: epidermal growth factor receptor; GSK-3*β*: glycogen synthase kinase 3 beta; APC: adenomatous polyposis coli; CK1: casein kinase 1; MMP-1: matrix metalloproteinase 1; MMP-7: matrix metalloproteinase 7.

A factor which may promote tumor genesis is represented by the mutations in tumor suppressor gene. APC gene is such an example; mutations occurring in this gene conduct to the synthesis of a short nonfunctional APC protein. This gene was identified in patients with familial adenomatous polyposis (FAP) as well as in patients with sporadic colorectal carcinomas [104–106]. Loss of heterozygosity (LOH) is a molecular instrument which identifies loss of an allele, by comparing the same region on a chromosome from normal DNA (heterozygote) with one from tumor DNA. LOH of APC gene was identified in many types of cancer including oSCC [107]. Studies have shown that APC protein induces the destruction of *β*-catenin (which is the factor that activates the transcription of oncogenes as Myc and Cyclin D1) and plays a role in microtubule assembly (see Figure 2). It was observed that in normal epidermis APC localization was only cytoplasmic while in SCC samples (tumoral cells and normal surrounding tissue) APC staining was negative for cytoplasmic localization but the nuclear staining was positive, which can help conclude that APC protein is present in the nucleus of proliferating cells [65]. The fact that APC nuclear staining was found in apparently normal cells surrounding SCC may be proof that this tissue was exposed to genetic changes that modified the APC expression, but in order to assert that this result is not normal, skin samples from patients with SCC in non-sun-exposed sites and from age-matched individuals without skin cancer should be examined [108].

The development of cSCC is influenced by many other modifications induced by UV radiation such as the presence of melanocortin-1 receptor (associated with fair skin and red hair) which represents a risk factor for developing cSCC as well as melanoma [109], increased telomerase activity which may protect cSCC from apoptosis [110], and mutations of NOTCH genes, which are tumor suppressor genes identified in 75% of patients diagnosed with cSCC [56].

Studies of molecular markers reflecting initial changes in skin carcinogenesis showed that, in sun-exposed skin, in which AK or SCC develops, the main molecular mutation is of gene p53. Considering the fact that this mutation is found in AK as well as in SCC represents the proof that this alteration is produced early in the development of cancer (AK is considered a precursor of SCC) [111, 112]. The fact that this gene is inactivated creates the perfect conditions for simple and numerous mutations to appear; this is the reason why cSCC is considered to have the highest mutation rate.

#### 4.1.3. Markers of Tumor Progression and Aggressiveness

Using reverse phase protein microarray (RPMA) samples from normal skin, AK, nonadvanced SCC, and advanced SCC were analyzed in order to identify which pathways were activated in the progression of SCC. The study showed that UV radiation activates numerous signal transduction pathways, such as p38, MAPK, and PI3K-AKT. These alterations may further influence apoptosis, proliferation, inflammation, and differentiation which may result in SCC development. It was demonstrated that in samples of skin from SCC and AK the percentage of phosphorylated AKT was significantly higher than in normal skin and in skin samples from metastatic SCC this protein value was the highest. The same results were obtained for mTOR (Ser2448), 4EBP1 (Ser65), 70S6K1 (Thr421), p70S6K1 (Thr421/Ser424), and S6 (Ser6) [55].

It appears that the inhibition of squamous cell differentiation is the most important mechanism that increases the invasiveness of cSCC; thus identifying molecules that can counteract this mechanism may help instate a more efficient treatment [63, 113]. S100 represents a family of calcium modulated proteins which include S100A7 (psoriasin) which was identified in keratinocytes harvested from psoriatic skin. It was noted that high concentration of S100A7 was found in various types of SCC (lung, oral cavity, bladder, and skin) which may indicate that this protein is a common biomarker for SCC [114] and it seems that this protein has an important role in metastasis [66–70]. It is believed that S100A7 may be involved in cell differentiation considering the fact that the more differentiated the cell population is, the higher the expression of S100A7 is. Also the gene that encodes the information necessary for the synthesis of this protein is located in chromosome 1q21 which contains the epidermal differentiation complex [115]. Studies have shown that S100A7 expression* in vivo* and* in vitro* can be influenced by induction and proliferation therefore S100A7 + cells switch to S100A7 − when the inducer is removed. It was noted that overexpression of S100A7 increased cell proliferation, survival rate, and tumor growth and cell differentiation was decreased, but when S100A7 expression was low cell differentiation markers increased while proliferation was inhibited [114].

The link between aggressive SCC and type VII collagen (Col7) is debated considering the fact that mortality is high (more than 78%) in patients with severe generalized recessive dystrophic epidermolysis bullosa (RDEB) from metastatic squamous cell carcinoma. Mutations occurring in COL7AI, the gene which encodes the information for Col7 synthesis, cause RDEB. This disease is characterized by skin and mucosal fragility due to a decrease in Col7 formation (the main component of anchoring fibrils) which leads to blister formation and chronic skin traumatisms (risk factor for SCC) [71]. Considering the fact that patients with dominant dystrophic epidermolysis bullosa (1 normal COL7A1 allele which means 50% normal Col 7 formation) develop SCC less than RDEB patients [71], scientists are trying to increase Col7 formation through divers methods (gene, protein, and cell therapy), but it was observed that high levels of Col7 are correlated with an important activation of the phosphoinositide 3-kinase pathway that leads to an increased invasion in SCC keratinocytes; therefore this kind of therapies should be applied with caution [116]. Matrix metalloproteinases are molecules implicated in maintaining homeostasis of many tissues including skin, by proteolysis of extracellular matrix. It was noted that MMP-7 has an increased concentration in cSCC (see Figure 2) but in cSCC that develops in patients with RDEB it has an even higher value which pleads for the aggressiveness of cSCC in this kind of patients [72–74].

It was discovered that SCC in mice is determined by Pam212, a keratinocyte cell population which does not have the ability of metastasizing, although cells that drift from Pam212 (LY lines) were found in lymph nodes metastases [117, 118]. Studies show that only LY lines can express keratin 8 (Krt8) and keratin 18 (Krt18) which are found in nonstratified epithelia but not in keratinocytes [119]. Cells from nonmetastatic and metastatic transformed keratinocytes were analyzed and it was discovered that Krt8 and Krt18 were linked together forming filaments and they were also in high concentration in metastatic cells [75]. The high invasiveness and potential for metastasis were demonstrated* in vitro* and they seem to be very strong in the population of cells that express both Krt8 and Krt18. This idea is supported by a study which concluded that a cell population which highly expressed Krt18 became metastatic only after overexpressing exogenous Krt8 [120]. Other studies show that coordinated coexpression of these two keratins reduce the metastatic potential of the tumor cells [119, 121].

Tyrosine kinase receptor family contains a larger group of receptors named erythropoietin-producing hepatocellular (Eph) receptors divided into two smaller subclasses A and B. The molecule that serves as ligand is ephrins [122, 123]. It was demonstrated that EphA subfamily is tumor suppressors, considering the fact that low concentration of EphA1 was identified in nonmelanoma skin cancer and low levels of EphA2 favour the development of chemically induced skin cancer in mice [124]. In cSCC, EphB2 determines proliferation, migration, and invasion, thus becoming the object of possible targeted therapies [125]. Farshchian et al. [125] identified high levels of EphB2 in primary and metastatic cSCC cells through microarray, qRT-PCR, and next-generation sequencing. These receptors were found on the surface of cSCC cells (clustered and bound with their ligands) as well as in the cytoplasm [125, 126]. Cells from normal cSCC, normal skin, and premalignant lesions were analyzed and it was shown that the concentration of EphB2 rises as normal cells progress to cancerous cells, which highlights that EphB2 overexpression is a process that starts early in cSCC development and has an important role in its invasiveness. To support this statement Farshchian et al. [125] showed that lowering EphB2 expression determined the inhibition of proliferation and migration of cSCC cells. These results identified EphB2 as a biomarker for cSCC progression and a potential therapeutic target [125].

#### 4.1.4. Cancer Stem Cells Biomarkers

Cancer stem cells (CSC) represent a population of cells with the unique characteristic of being solely responsible for initiating and maintaining tumor growth [127, 128] (Tables 1, 2, and 3). Therefore, it is very important to identify any kind of biomarker related to CSC, which may provide vital information such as risk of metastasis, resistance to therapy, and recurrence. There are studies [96, 129] that investigated the role of CD133 (CSC biomarker), a transmembrane glycoprotein present in normal hematopoietic stem cells responsible for proliferation and differentiation in various types of cancers, including skin cancer, demonstrating that overexpression of this protein is correlated with poor prognosis [46, 76–81]. Samples of cSCC tissue were analyzed and it was observed that increased expression of CD133 was correlated with low differentiation and advanced tumor stage. Studies have shown that CSC CD133 + are resistant to apoptosis induced either by transforming growth factor *β* or by tumor necrosis factor and the self-renewal capacity of this cells is lost once CD133 is lost [130]. New treatment strategies that target CD133 would be useful for patients with high expression of this protein, who are at risk of developing cSCC with poor prognosis [131].

#### 4.1.5. Molecular Therapeutic Targets

In the last decades cancer therapy studies have focused on targeted molecular treatments (monoclonal antibody, small molecule tyrosine kinase inhibitor); therefore scientists have developed a great interest for EGFR (epidermal growth factor receptor) which is a tyrosine kinase receptor and two of its most important ligands are epidermal growth factor and transforming growth factor-*α* and its roles are skin cell proliferation and differentiation, thus contributing to tumorigenesis [57–59]. Studies show that EGFR has high values in many types of cancer (oropharynx, oesophagus, stomach, colorectal, pancreas, non-small cell carcinoma of the lung, and SCC) [132]. The mechanism explaining why EGFR has high expression in HNSCC is not completely elucidated, although several hypotheses including mutations in the receptor, high ligand levels, and increased mRNA transcription have been proposed (see Figure 2) [59]. However, the fact that monotherapy with EGFR inhibitors was not as successful as expected makes researchers believe that EGFR might not be the main component in the oncogenic process [133–135]. Studies show that only 47% of metastatic disease in cSCC overexpress EGFR which leads to the hypothesis that the metastatic cell population that does not overexpress EGFR may originate from another clone, hypothesis supported by the fact that a study on the use of EGFR inhibitor (gefitinib) in patients with metastatic cSCC showed that the therapy had no results [136].

### 4.2. Oral SCC

The incidence of* oral SCC* has a wide variability worldwide depending on food and lifestyle habits (alcohol and cigarette). In order to diagnose in an early stage this type of cancer, scientists tried to find new biomarkers that could provide the opportunity of predicting the prognosis. Therefore they studied cytokeratin 19, which is one of the 20 cytokeratin polypeptides discovered (they are structural proteins involved in epithelial intermediary filaments formation). Cytokeratin 19 is expressed by normal cells as well as by some cancerous cells like lung cancer cells [82, 137]. CYFRA 21-1 is the serum soluble component of this cytokeratin and its high values was linked with high mortality in patients with lung cancer [138, 139]. Extrapolating, scientists observed that patients with HNSCC and high concentrations of CYFRA 21-1 had a poorer prognosis because it is considered that this molecule is released in the blood stream by metastatic tumor cells [82–88].

Another molecule that can help identify patients with a high mortality risk is CRP (inflammation marker), the low survival rate and cancer invasiveness being demonstrated for oSCC (inflammation provides the circumstances for proliferation and angiogenesis); also studies showed that elevated CRP was correlated with bone, skin, and lymph node invasion [140, 141]. Scientists tried to link these two biomarkers (CRP and CYFRA 21-1) in order to see if a prediction for poorer prognosis could be made before surgery and found that patients with increased concentrations of both of them were at a higher risk of developing distant metastases. As established above, tumor cells release CYFRA 21-1 into the blood stream; there they activate inflammatory cells that release inflammatory cytokines which in the end increases the CRP serum value [142].

In oral squamous cell carcinoma, measuring mitochondrial DNA (mtDNA) may be useful for postoperative monitoring considering the fact that an important number of patients with head and neck SCC (HNSCC) that had histological negative margins had mtDNA mutations [89, 90]. It is necessary to determine this molecule quantitatively, because it was demonstrated that even though there were no identifiable metastasis, high mtDNA values were detected in the organs and blood of mice injected with Sa3 cells (cutaneous squamous cell carcinoma cells). Uzawa et al. analyzed postoperative blood samples from 61 patients, and of those 16 had high mtDNA mutations which were correlated either with a local recurrence or with distant metastasis within the next 9 months after surgical treatment [143]. It is really important to note that mutations in mtDNA are identified only in tumoral tissue, and depending on the intensity it can be used as a prognostic predictor for patients with oSCC. Although low mutant mtDNA detection could not be correlated with the fact that the more the phenotype is differentiated, the better the prognosis is, this biomarker has a great potential of becoming a criteria of including a patient in high/low risk group even though histologically they are tumor-free [144].

### 4.3. Genital SCC

*Penile cancer* is a rare condition and its incidence was linked to lack of circumcision and hygiene, phimosis, HPV infection, and tobacco use [144–147]. Viral infection is a very important risk factor; HPV DNA incorporates itself in the human genome and induces an important expression of viral genes E6 and E7 which inactivates tumor suppressor genes. This type of cancer is another example of the necessity of reliable biomarker that can predict the prognosis, considering the fact that for the moment inguinal metastases are the most important prognostic factor [148]. Recently a study was conducted on 20 patients divided into 2 groups: group 1 composed of patients diagnosed with HPV and PSCC (penile squamous cell carcinoma) and group 2 (control group) containing samples of foreskins from patients with HPV and without tumors [148]. After analyzing the samples from the two groups the results showed that in group 1 the concentration of Hsp70 was very high and considering the fact that this protein was also found in high concentration in other types of cancer, it is presumed that it may help tumorous cells survive apoptosis and necrosis. This protective role was demonstrated by the fact that if/when an adenovirus expresses anti-Hsp70 it leads to an important tumor cell death in breast, colon, prostate, and liver cancer [91]. In group 1 component C3 of complement was not detected and a theory that may explain why this result occurred is the fact that viral proteins have the ability of counteracting the immune response; therefore viral infection has a protective role over the tumor cells providing them an environment favourable for their development [149]. Other molecules studied by [150] are plakins, which represent a family of molecules which form the links between filaments, desmosomes, and hemidesmosomes and plectin is a cytolinker of this family. Studies showed that defective expression of plectin induces genomic instability which creates favourable circumstances for cancer development and progression [92].

*Vulvar SCC* accounts for more than 90% of the malignant tumors with this localization [151]. Emerging evidence suggests the existence of two separate entities regarding the development of epidemiological, pathological, and clinical characteristics of vulvar SCC, namely, one associated with human papilloma virus infection (HPV) and a second independent of HPV. In trend with recent efforts for surrogate biomarker discovery in cancers [30–33, 35, 54, 152–155], research of vulvar SCC has demonstrated the importance of detecting differentially expressed proteins for early diagnosis and timely therapeutic intervention. In this regard, numerous studies indicate that* p16 expression* indicate a less aggressive variant of vulvar SCC, less likely to recur and with no related deaths. By contrast, patients with* p53 expression* had a poor prognosis and significantly increased local recurrence and disease-specific mortality [156]. Other molecular markers with negative impact in patients with vulvar SCC include* cofilin-I*,* galectin-7, and wee1*.* Cofilin-I* expression was found to be significantly increased in vulvar SCC compared with normal tissue and was suggested to be involved in vulvar carcinogenesis and subsequent tumor progression [93]. Downregulation of* galectin-7* and high* wee1* expressions was found to correlate with advanced clinical stage, poor tumor differentiation, and regional lymph node metastasis [94, 95]. Moreover, a gradual reduction into disappearance of estrogen-related receptor-*α* expression was observed from healthy vulva to precursor lesions and further to SCC [157]. Among these,* cofilin-I* was proposed as a potential target alone for therapeutic intervention as* cofilin-I* silencing by siRNA significantly reduced cell progression in vulvae SCC [93].

## 5. Conclusions

Considering the significant risk of recurrence and metastasis of SCC, there is a high necessity to discover novel molecules harvested from various biological samples that could explain the occurrence and evolution of this keratinocyte-derived tumor. In this regard, protein-focused research based on high-throughput proteomic technologies has evolved rapidly to identify unique biosignature of skin cancer.

Analyzing differences between normal, inflammatory, and malignant keratinocyte proteome holds special promise for novel biomarker discovery in SCC that could be used in the future for early detection, risk assessment, and tumor monitoring. Furthermore, identification of novel potential biomarkers for SCC development and progression will aid the discovery of individualized targeted therapies for these patients.

## Figures and Tables

**Table 1:** Available treatment options for skin SCC [54].

Nr. crt.	Type of cSCC	Therapy	Adjuvant
(1)	Low risk cSCC	Electrodessication Curettage	

(2)	Invasive cSCC	Surgical excision Mohs micrographic surgery	Radiation therapy provides good locoregional control and can also be used as primary therapy for lesions that cannot be surgically excised EGFR inhibitors

(3)	Metastatic cSCC		Chemotherapy

(4)	Prevention	Decreased UVR exposure Correct and early treatment for precancerous skin lesions	

**Table 2:** Molecular pathways governing epidermal stem cells homeostasis and tumorigenesis.

Nr crt	Molecular pathway	Roles
(1)	p63	Proliferation, self-renewal, development, morphogenesis, tumorigenesis

(2)	SRF/MAL	Differentiation, development, cytoskeletal regulation

(3)	mTOR	Senescence, cell size, tumorigenesis oxidative stress [55]

(4)	p75	Apoptosis, communication, differentiation

(5)	Hippo	Organ size, antiproliferative, apoptosis maintenance, antitumorigenic

(6)	Notch	Differentiation, morphogenesis, suprabasal switch [56]

(7)	FOXM1	Proliferation, genome instability, tumorigenesis

(8)	p38 MAPK	Proliferation, wound healing, differentiation, cell migration, invasivity, tumorigenesis [55]

(9)	BMP	Proliferation, differentiation, plasticity, wound healing

(10)	TGF*β*	Proliferation, immortalization, tumorigenesis [38, 41–44]

(11)	TGF*α*	Proliferation, hyperplasia, immortalization, tumorigenesis

(12)	EGFR	Proliferation, maintenance, tumorigenesis [57–59]

(13)	c-myc	Proliferation, differentiation, tumorigenesis

(14)	Shh	Development, morphogenesis, proliferation cell survival

(15)	Wnt	Proliferation, self-renewal, wound healing, morphogenesis, tumorigenesis

**Table 3:** cSCC biomarkers.

Nr. crt.	Biomarker	Roles
(1)	CFH FHL-1 Complement factor 1	(i) Inhibiting one of the three pathways that activate the complement C3 (ii) Facilitating progression and migration of cSCC cells [60–62]

(2)	Serpin A1	(i) Coagulation (ii) Inflammation (iii) Turnover of extracellular matrix (iv) Inhibiting natural killer cell activity (v) Stimulating malignant cell proliferation but not normal skin cell proliferation (vi) Antiapoptotic effect [63, 64]

(3)	APC	(i) Inducing the destruction of *β*-catenin (ii) Having a role in microtubule assembly [65]

(4)	Phosphorylated AKT, mTOR (Ser2448), 4EBP1 (Ser65), 70S6K1 (Thr421), p70S6K1 (Thr421/Ser424), S6 (Ser6)	(i) Influencing apoptosis, proliferation, inflammation, and differentiation [55]

(5)	S100A7	(i) Role in metastasis [66–70]

(6)	Col7A1	(i) Encoding the information for Col7 formation [71]

(7)	MMP-7	(i) Maintaining homeostasis of many tissues including skin, by proteolysis of extracellular matrix [72–74]

(8)	Krt8 Krt18	(i) Together they induce a higher rate of invasiveness in a cell population [75]

(9)	CD133	(i) Proliferation (ii) Differentiation [46, 76–81]

(10)	CYFRA 21-1	(i) Component of structural proteins involved in epithelial intermediary filaments formation [82–88]

(11)	mtDNA	(i) Mitochondrial functions [89, 90]

(12)	Hsp70	(i) it is presumed that it may help tumorous cells survive apoptosis and necrosis [91]

(13)	Plectin	(i) Cytolinker of plakins family which forms the links between filaments [92]

(14)	*Cofilin-I*	(i) Vulvar carcinogenesis (ii) Tumor progression [93]

(15)	*Galectin-7* *wee1*	(i) Invasiveness (ii) Poor tumor differentiation [94, 95]

(16)	EphB2	(i) Determining proliferation, migration, and invasion [96]
